# A Systematic Review of the Contribution of Dance Movement Psychotherapy Towards the Well-Being of Children With Autism Spectrum Disorders

**DOI:** 10.3389/fpsyg.2021.719673

**Published:** 2021-10-08

**Authors:** Supritha Aithal, Zoe Moula, Vicky Karkou, Themis Karaminis, Joanne Powell, Stergios Makris

**Affiliations:** ^1^Research Center for Arts and Wellbeing, Edge Hill University, Ormskirk, United Kingdom; ^2^Faculty of Health, Social Care and Medicine, Edge Hill University, Ormskirk, United Kingdom; ^3^Faculty of Medicine, School of Public Health, Imperial College London, London, United Kingdom; ^4^Department of Psychology, Edge Hill University, Ormskirk, United Kingdom

**Keywords:** systematic review, dance movement psychotherapy, autism spectrum disorder, meta-synthesis, intervention

## Abstract

**Background:** The present review provides an original examination of published literature on the use of Dance Movement Psychotherapy (DMP) as an intervention for children with an Autism Spectrum Disorder (ASD).

**Method:** The review was systematically conducted using the Preferred Reporting Items for Systematic Review and Meta-Analysis (PRISMA) guidelines. A protocol consisting of four phases: identification; screening and selection; data extraction and synthesis; quality assurance was developed and registered with the PROSPERO. A search strategy was developed using population and intervention as the key concepts and ten databases were searched between 6.1.2018 to 4.4.2018 and 10.07.2021 to 20.07.2021. The intervention characteristics were extracted based on the TIDieR template for intervention description and replication checklist. Quality assessment and level of evidence of all the included studies were evaluated using the Mixed Methods Appraisal Tool (MMAT) and the Centre for Evidence-Based Medicine (CEBM) for treatment criteria.

**Results:** Nine research studies with a total of 133 participants were identified through a systematic search process. There was only one mixed-methods study with the component of randomisation found during the literature search. Collected information was synthesised in relation to (a) ways in which dance movement psychotherapists work with children; (b) data collection methods and findings. Results from the reviewed literature suggest that DMP can potentially promote various aspects of well-being in children with ASD. Eight out of nine studies mentioned the effects of DMP on improving different social and communication skills. However, results from quality assessments and synthesised outcomes indicate that research in DMP is still in its infancy.

**Conclusions:** We conclude that further large-scale, high-quality studies are required to generate further evidence that explains the processes involved in DMP, the effectiveness of DMP, the relationship between therapeutic factors of DMP, and research findings for children on the autism spectrum.

**Systematic Review Protocol Registration:** PROSPERO, identifier: CRD42018087912.

## Introduction

The number of individuals diagnosed with ASD have increased in the last decade with 1 in 160 individuals being diagnosed with Autism Spectrum Disorders (ASD) worldwide (Elsabbagh et al., 2012). This figure is found to be even higher in some areas of the developed world reaching 1 in 100 in the United Kingdom (National Autistic Society, 2020) and 1 in 59 children in the United States of America (USA) according to estimates from Centres for Disease Control and Prevention (Christensen et al., 2018). The well-being of such individuals is often challenged due to persistent difficulties in social interactions, communication, as well as restricted and repetitive behaviours and interests (Irwin et al., 2011). The impact these issues have on family members and carers is often major: having a child with ASD in the family can be demanding, time-consuming and expensive (Jordan and Jones, 1999; Green et al., 2006). It therefore comes as no surprise that caregivers reportedly seek alternate interventions to support their children. Effective treatments, however, remain elusive (Canitano and Bozzi, 2015) despite a clear need for them. Most often, available interventions focus on teaching socially acceptable norms or imposing socially acceptable communication modes on children rather than value inherent differences (Nind, 1999; Desforges and Abouchaar, 2003; National Autistic Society, 2020). In addition, most of the available interventions for children with ASD follow behavioural approaches which rely on drill-like activities that encourage repetition of specific skills with constant instructions or teacher direction. These interventions do not typically involve structured reflection or intuitive retorting (Silberman, 2015; Baron-Cohen, 2017; Mottron, 2017). It is therefore less common for available interventions to focus on strengths and attempt to reach children where they are at, listening and responding to children's specific needs (Mottron, 2017).

Multi-dimensionality in the concept of well-being plays a role in understanding the well-being of children with ASD. Acknowledging the complexity, plentiful dimensions and viewpoints, the present study bases its definition of wellbeing on the publication of Dodge et al. (2012). Here well-being is viewed as the balance point between an individual's resource pool and the challenges faced. Dodge et al.'s (2012) definition is based on the principles of equilibrium/homeostasis and the fluctuating state between challenges and resources. Therefore, the way children with ASD dynamically utilise their resources to address the numerous emotional, social and communication challenges they encounter in life to maintain the balance is viewed as well-being in this context.

In the UK, National Institute for Health and Care Excellence (NICE, 2016) guidelines for ASD in under 19s recommends that children and young people with ASD must have access to multidisciplinary health and social care services including mental health. The general principles of care and specific interventions for the core features of ASD consider play-based strategies, behavioural and developmental models. For mental health challenges, group/individual cognitive behavioural therapy (CBT), group non-directive supportive therapy (NDST), group/family-based interpersonal psychotherapy (IPT), psychodynamic psychotherapy are recommended depending on the severity of the issue. As the guidelines are from 2016, there might have been new studies on the effectiveness of arts-based interventions that need to be aknowledged. Thus, a new systematic review on these studies is warranted.

### Description of the Intervention

Dance/Movement Therapy, or Dance Movement Psychotherapy^1^ (DMP) as it is known in the UK, is a psychotherapeutic approach that aims to support the integration of mind and body through the use of creative movement expression (American Dance Therapy Association, 2018; Association for Dance Movement Psychotherapy UK, 2020). Within DMP, the body is viewed as a container of experiences which can be communicated through movements. DMP is used with a wide range of client populations in several settings and is offered by qualified practitioners who, in the UK, undergo Master's level training for a minimum of 2 years (Association for Dance Movement Psychotherapy UK, 2020). Methods such as rhythmic circle dance formations, group or dyadic improvisation and expressive movement processes are tailored to the needs of the individual or group (Levy, 1988; Meekums, 2002; Payne, 2003). In DMP, movement is viewed as symbolic representation and as evidence for both conscious and unconscious processes (Meekums, 2002; Karkou and Sanderson, 2006). The interdependence between movement and emotion (Bernstein, 1975; Rossbeg-Gempton and Poole, 1992) is thought to enable the unconscious to unfold (Levy, 1988; Fischman, 2001) thereby promoting health and growth toward personal well-being (Fischman, 2001). Some of these ideas are also relevant to working with children with ASD (Karkou, 2010).

DMP interventions for children with ASD focus on body-informed and non-verbal interpersonal exchanges that attempt to meet the children empathetically (Adler, 1968; Siegel, 1973; Kalish, 1977; Erfer, 1995; Loman, 1995; Parteli, 1995; Torrance, 2003; Tortora, 2005; Scharoun et al., 2014). Mirroring and other techniques that enable kinaesthetic empathy in ASD populations are commonly stated in clinical practice reports as ways of supporting non-verbal relationships (Tortora, 2010; Wengrower, 2010; Devereaux, 2012; Martin, 2014). This technique is similar to those used in the autism field such as intensive interaction (Nind and Hewett, 1988), where the value of meeting the child non-verbally is acknowledged. However, mirroring within the context of DMP practise does not refer to simply copying one's actions but also involves an affective attunement to the non-verbal presentation and movement preferences of the child (Meekums, 2002). Whilst mirroring and similar techniques are used extensively in DMP practise, the value of working in this way with children with ASD remains largely anecdotal with limited systematic evaluation.

### Rationale for Systematic Review

Although research in the field of DMP has shown an upward trend in the past two decades (Meekums, 2010), DMP remains a young profession that relies heavily on creative, subjective and clinical reports (Rova, 2017). When research methodologies are adopted, they use small samples, qualitative designs, and descriptive, phenomenological, experience-based approaches or case studies (Serlin, 1996; Behrends et al., 2012; Hervey, 2012), resulting in insufficient empirical evidence for the wider use of DMP. Indeed, compared to other practices, DMP has not been used widely with children on the autism spectrum. Green et al. (2006) reported that in the USA only 2.4 % of children on the autism spectrum participated in DMP in comparison to other interventions, while DMP stood in 55th position on a list of treatments used by parents. To make DMP interventions more widely available, further research is needed to empirically validate the effectiveness of the DMP as an intervention and to identify the most appropriate ways of working with ASD client populations. Such research will bridge the gap between evidence-based practise and practise-based evidence (Barkham and Mellor-Clark, 2003).

Earlier reviews in DMP and ASD have either been too generic (Takahashi et al., 2019) or have focused only on adults with ASD and their needs (Marchant et al., 2018; Shuper Engelhard and Vulcan, 2021). Information such as dosage (frequency and duration of DMP sessions), theoretical frameworks, therapeutic techniques and overall process used in DMP for children with ASD are yet to be synthesised. To our knowledge, no existing studies have documented explicitly the changes and outcomes during and/or after DMP intervention in children with ASD. Further research is required to systematically report on how DMP is practised and to evaluate the quality of the existing evidence on the contributions of DMP interventions for children with ASD. Therefore, this review aims to examine how the processes involved in DMP could support the development of a wellness toolbox to cope with the situation, and implement the tools when necessary.

### Research Questions

The present systematic review explores the following research questions:

1. How do dance movement psychotherapists work with children with ASD in terms of, theoretical frameworks, techniques, overall process and dosage in published research?
2. How do different studies examine the effectiveness and processes involved in DMP interventions? What are their findings?

## Methods

This integrative systematic review was based on the processes used in meta-analyses and qualitative evidence synthesis of Cochrane Reviews (Higgins and Green, 2011). Unlike Cochrane Reviews, however, the exclusive reliance on Randomised Control Trials (RCTs) was changed in this review to include other quantitative, qualitative and arts-based research studies on DMP for children with ASD. We also extended the meta-synthesis approach by including not only qualitative findings in the briefs but also quantitative studies. In order to combine both approaches, we adopted the principles of pragmatism (Haack and Lane, 2006), according to which all evidence available at the time is collected. The review was implemented using the Preferred Reporting Items for Systematic Review and Meta-Analysis (PRISMA) guidelines (Page et al., 2021) to ensure that the review was conducted systematically and that results were replicable.

Firstly, a protocol was developed and registered with an open access online database PROSPERO (https://www.crd.york.ac.uk/PROSPERO; Registration Number CRD42018087912). The protocol included the following stages:

**a) Identification:**
  The following electronic databases were searched during two time periods (6.1.2018 to 4.4.2018 and 10.7.2021 to 20.7.2021) to present the updated studies: Academic search primer; CORE; PsyARTICLES; Emerald Health and Social Care Journals; PsycINFO; Proquest Health Research Premium Collection; Wiley; PubMed; BioMed Central Journals; and Cinahl Complete. Additional hand searches in relevant journal databases and different universities' catalogues were also conducted.

**Search Formula**
  1. Step 1: Autis^*^ OR Asperger^*^ OR Rett^*^ OR “Pervasive Developmental Disorder^*^” OR “Neurodevelopmental Disorder^*^” OR “Childhood Disintegrative Disorder^*^”
    AND
  2. Step 2: “Dance movement therapy” OR “Dance/Movement therapy” OR “Dance Movement Psychotherapy” OR “Movement Therapy” OR “Movement Psychotherapy” OR “Authentic movement” OR “Primitive expression”

**b) Study Screening and Selection Process**
  The first author as part of the doctoral study ran the searches in various databases, identified relevant studies and removed duplicate titles using Zotero software 2018. Another PhD student (ZM) and the first author independently screened at the level of title and abstract based on the predetermined inclusion/exclusion criteria. Inclusion and exclusion criteria were also applied for full texts. In cases of missing data, the authors were contacted to provide original reports. Any cases that remained unclear were discussed with the director of studies (VK).
  With regards to eligibility (Table 1) to target studies that are relevant to DMP for children with ASD, we used a modified version of PICOS method (Bowling and Ebrahim, 2005). In particular, we decided eligibility based on four components: Participant Population-P, Intervention-I, Outcomes-O and Study design-S; but did not consider Comparison (C) as the review does not aim to compare DMP intervention with other types of interventions or groups without any intervention. The outcome component included studies oriented toward outcomes as well as studies describing the therapeutic processes to further understand the contribution of DMP for children with ASD.

**c) Data Extraction and Management**
Microsoft Excel and the specialised software package Covidence (2018) were used to organise and manage all relevant information from the studies. The data extraction focused on answering the research questions and included two main categories:

- *Ways in which dance movement psychotherapists* work with children with ASD. This category looked at theoretical frameworks, therapeutic techniques, overall process and intervention dosage. Theoretical frameworks referred to the wider concepts that informed the approach of DMP were documented. Therapeutic techniques considered the methods that therapists practically used during the session. The overall process recorded the DMP session structures. Finally, dosage referred to frequency, duration and intensity of DMP sessions. These intervention characteristics were extracted based on the TIDieR template for intervention description and replication checklist (Hoffmann et al., 2014).
- *Data-collection methods and findings* captured what type of assessments were administered, how the assessment took place and the changes noticed in the participants after attending DMP.

**d) Quality Assessment**
The Mixed Methods Appraisal Tool (MMAT) (Hong et al., 2018) was used to critically evaluate the quality and risks of bias in the reviewed studies and also to ensure that the studies were reviewed with equal rigour. The MMAT was chosen because it is specifically designed for the appraisal stage of systematic mixed studies reviews, and the five sub-sections cover the methodological quality appraisal of: qualitative research, randomised controlled trials, non-randomised studies, quantitative descriptive studies, and mixed methods studies. Further, the studies were evaluated as per the levels of evidence for therapeutic studies based on the criteria developed by the Centre for Evidence Based Medicine (CEBM) for treatment (March, 2009).

**Table 1:** Study selection criteria.

**Framework**	**Criteria**
Types of participants	Studies with participants of 16 years of age or below; diagnosed with ASD of any severity were considered for this review. Furthermore, studies in which the majority (>75%) of the participants were younger than 16 years or had a diagnosis of ASD were included
Types of interventions	Studies where DMP was delivered as an intervention by a qualified therapist (in the countries where training is available) with clear goals and therapeutic process were considered. All DMP approaches were considered even if they were delivered alongside other arts therapies or as a combination with other arts therapies. However, studies where dance training or other types of recreational dance programmes without a psychotherapeutic process were not included in this review
Types of outcome measures and processes	Outcomes of DMP on social, cognitive, emotional, behavioural, physical, academic measured through standardised measures were considered. Methods that captured the perspectives of children, parents, therapists, and teachers on both the process and the outcomes were included. Video analysis methods that looked at therapeutic process were also considered. Studies that neither investigated the therapeutic process nor its outcomes were excluded
Types of studies	Any type of empirical research (quantitative, qualitative, mixed, or arts-based methods) were included. Studies not included were: non-empirical research studies (e.g., secondary sources, opinion-based, editorials, policy reviews and statements, commentaries), studies not published in English, unpublished Master level dissertations, unpublished conference presentations, conference proceedings where full-length articles are not available, clinical case examples without rigorous research methodology and narrative articles and reviews without rigorous research methodology

## Results

**Figure 1:**
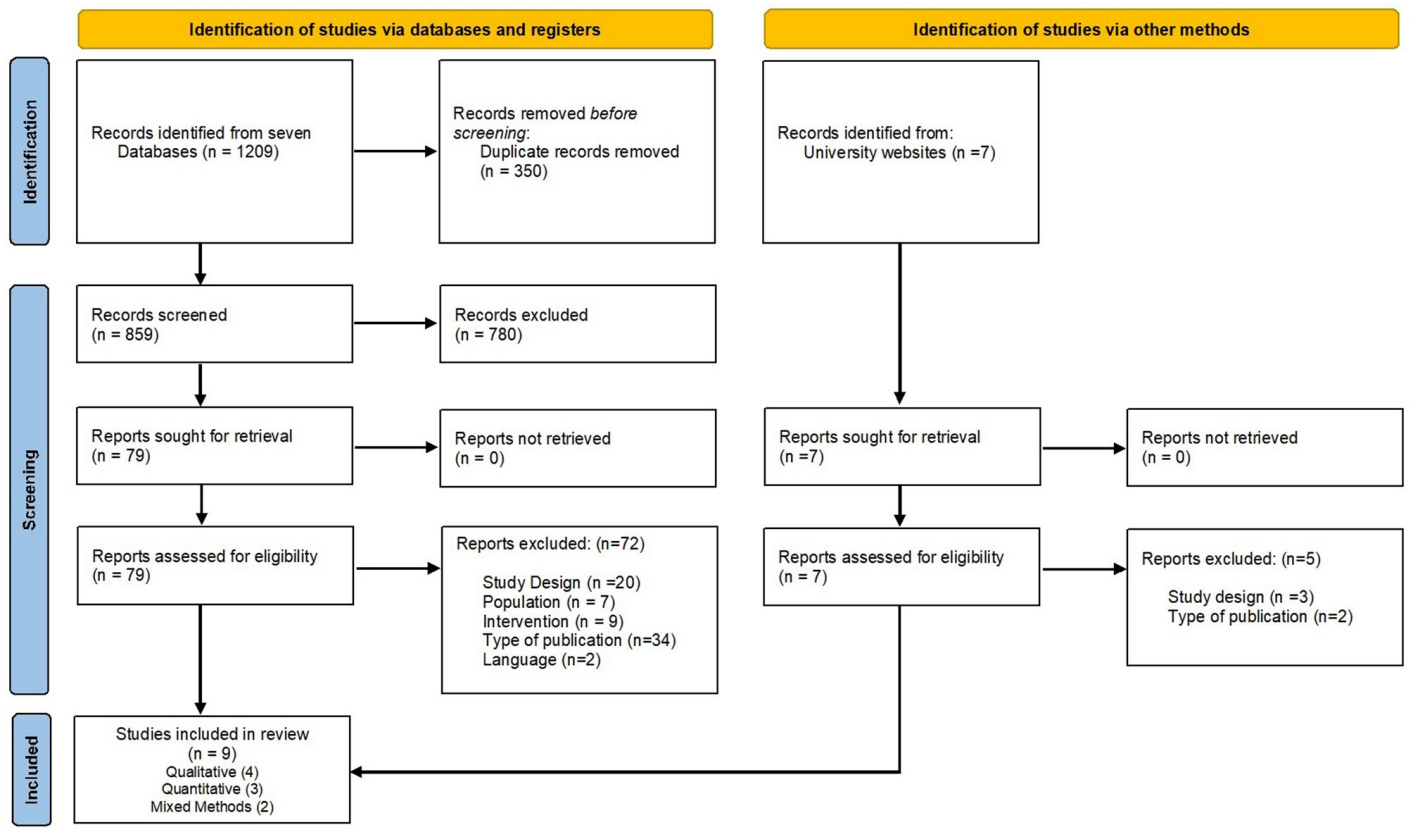
PRISMA flowchart diagram. From Page et al. (2021). For more information, visit: http://www.prisma-statement.org/.

As shown in the PRISMA flow chart (Figure 1), 1,209 records were identified from searching across seven databases, out of which 780 were irrelevant and were treated as noise. Eight hundred and fifty nine articles were screened at the title and abstract level. Handpicked searches from various universities' repositories and correspondence with researchers generated 7 relevant records which were considered for the full-text screening. Eighty eight records (79 from the databases and 7 from handpicked searches) were evaluated for the full-text eligibility. In total, nine studies met the inclusion criteria for the data extraction process. One of the doctoral studies included in the review contained four sub-sections (Samaritter, 2015) which involved different participants. However, only those three sub-sections which met the inclusion criteria were counted as one study during data extraction and synthesis.

### Overview of Included Studies

The nine studies included in the review are summarised in Table 2. With regards to the research questions that are addressed, some studies (Samaritter, 2015; Athanasiadou and Karkou, 2017) focused on the process of DMP while others described either the intervention techniques (Wengrower, 2010) or ways of evaluating practice (Houghton and Beebe, 2016); the remaining studies focused on outcomes (Hartshorn et al., 2001; Chiang et al., 2016; Aithal, 2020; Sengupta and Banerjee, 2020).

**Table 2:** Study characteristics.

**Author and year**	**Country**	**Participants (sampling size, diagnosis, age)**	**Study design**	**Methods of data collection**	**Findings**	**Level of evidence [CEBM, March 2009]**
Aithal (2020)	UK	*N* = 26, all with ASD; Age range = 8–13 yrs Mean Age = 10.65 yrs	Mixed methods cross over design	• Questionnaire • Semi structured interview • Arts based methods	• Significant improvements in social communication questionnaire (SCQ) • Minimally clinically important differences in strengths and difficulties questionnaire and SCQ • Three qualitative themes and 18 sub themes reflecting therapeutic process of change • Artistic inquiry identified six key moments of change	2B
Sengupta and Banerjee (2020)	India	*N* = 3, all with ASD, Age range = 3–11 yrs	Multiple case studies	Pre-post design case study	• Improvement in body attitude checklist and communication • Effects of DMP were sustained in three cases during post interventions assessments (3 and 6 months) • The effects of DMP declined after 9 months of intervention	4
Athanasiadou and Karkou (2017)	UK	*N* = 3; all with ASD; Age range = 6–7 yrs	Series of case studies (Arts-based)	• Video Recordings • Somatic responses (including drawings of a body figure, and written and video recordings) • Written reflections	• Enhanced social bonding and relationships • Increased expressive and receptive vocabulary • Improved self-regulation and empathy • Reduced stereotypical behaviours	4
Devereaux (2017)	USA	*N* = 17; 15 children with ASD, one child with down syndrome and one child with cerebral palsy; Age range = not mentioned	Interpretive qualitative approach	Person to person Semi-Structured Interviews	• Building connexion, awareness with self and others • Improved regulatory behaviour, coping skills • Enhanced capacity to focus, regulate energy levels and relax	4
Houghton and Beebe (2016)	USA	*N* = 1; ASD; Age = 6 yrs	Video micro analysis	• Video Microanalysis • Narrative of the first 80 s based on real time and slow motion	Micro disruptions of the connexions, missed opportunities for connexions, critical points in interactions were identified	4
Chiang et al. (2016)	Taiwan	*N* = 34; all with ASD; Age range = 2–4 yrs	Quasi-experiment research design	• Pre- and post-intervention and 3 month follow-up • Semi-structured observations	• No significant change in joint attention and engagement immediately after the intervention • Improvement during 3 months follow up in engagement state, supported joint engagement (child initiated) and co-ordinated joint engagement (child initiated)	2C
Samaritter (2015)	UK and Netherlands	*N* = 4; All with ASD; Age range = 6.3–17.2 yrs Mean Age = 12.02 yrs	Study (1) Mixed-methods design: Retrospective video analysis	Retrospective movement annotation and analysis of video vignettes of interpersonal relating in dyadic DMP	• Development of Social Engagement and Attunement Movement (SEAM) scale, with overarching themes (space, time, weight) and specific movement categories • Individual profiles of all four cases showed an increase within SEAM categories and an overall increase of the number of SEAM markers that could be recognised in the interpersonal movement actions	4
			Study (2) Mixed-methods design: Retrospective video analysis	Contents analysis of the therapist's actions	∙ Four basic themes were identified for structuring DMP sessions for ASD and illustrated with examples a. Procedural structure of the therapy process b. Structure of the sessions c. Relational modes d. Movement actions	4
		*N* = 4, all with ASD Age range = 11.9–17.1 yrs Mean Age = 14.9 yrs	Study (3) Mixed-methods design	Replication of Shared Movement Approach (SMA) intervention and outcome evaluation with SEAM scale	Average pre-post-intervention outcomes for group of four showed a positive trend on youth self-report scale, social responsiveness scale, and child behaviour checklist	4
Wengrower (2010)	Spain	*N* = 3 (2 children with ASD and one child with PDD); Age range = 3–8 yrs; Mean Age = 6.3 yrs	Multiple case study design	Narratives of three DM therapists as they wrote then in their case study, therapy journals, treatment reports	• Enhanced therapeutic relationship that implies a sense of mutuality, attraction and interest to know each other better • Created a shared and translation playing space where bonding evolved	4
Hartshorn et al. (2001)	USA	*N* = 38, all with ASD; Age range = 3–7 yrs; Mean Age = 5 yrs	Experimental design	• Video recording • Behaviours were coded and observed during the first and last movement sessions.	• Statistically significant reduction in resistance to teacher, negative response to touch and wandering behaviours • Significant improvement in on -task passive behaviour • No difference in eye contact, social relatedness, on–task active behaviours and stereotypical behaviours	2C
**Summary**						
Year range: 2001–2020	Countries: EU, UK, USA, Taiwan and India	*N* = 133; Mean age= 9.1 yrs (age not mentioned in one study)	QUAL-4 QUAN-3 MIXED-2	Observing video recordings of the sessions is the most common method-5 studies, followed by semi-structured interviews and questionnaires −2 studies	Improvements were observed in • Group connexions • Relationship with the therapist • Awareness of self and others • Emotional regulation • Coping mechanisms • On-task behaviours	

*EU, Europe; UK, United Kingdom; USA, United States of America; N, Number of Participants; QUAL, Qualitative; QUAN, Quantitative*.

The most recent of the nine studies included in this review was published in 2020 and the oldest in 2001. The majority were from the West (USA, UK, and EU); one study was from Taiwan and one from India. Special education schools were the most common environments for DMP sessions to take place followed by clinical or hospital-based settings. In total, there were 133 participants across these nine studies. Sample sizes were small ranging from one to thirty-eight participants. Hartshorn et al. (2001), with an experimental research design, had the largest sample size: 38 participants and the second largest sample was in the Taiwan-based study (Chiang et al., 2016) which included 34 participants (18 in the experimental condition). Houghton and Beebe's (2016) video micro-analysis study involved an individual case that used small videoclips of the therapist and participant interaction from a session.

In eight out of the nine studies, the participants were children with a formal diagnosis of ASD, albeit the severity of ASD presented remained unclear. Studies with more than 75% children or adolescents of 16 years of age or below were included for the review. From the studies included, the average age of the participants was 9.48 years (age range = 2–17.2 years). An exception to these studies was the one by Devereaux's (2017), which involved 13 special educators reporting on their observations of DMP sessions for children with ASD. This study was included in the review because it described the contribution of DMP for children on the autism spectrum from the educators' perspective and answered one of the review research questions exploring the findings of DMP intervention for children with ASD.

The nine studies included in the review followed different methodological approaches; four were qualitative (Wengrower, 2010; Houghton and Beebe, 2016; Athanasiadou and Karkou, 2017; Devereaux, 2017) and specifically, one of which followed an arts-based research design (Athanasiadou and Karkou, 2017). There were three quantitative (Hartshorn et al., 2001; Chiang et al., 2016; Sengupta and Banerjee, 2020) studies. The doctoral studies of Samaritter (2015) and Aithal (2020) were conducted using a mixed-methods design.

### Research Question 1- Ways in Which Dance Movement Psychotherapists Work With Children on the Autism Spectrum

To address the first research question, we extracted information on therapeutic frameworks, techniques, overall processes that informed DMP intervention and the dosage in which the sessions were delivered (Table 3).

**Table 3:** Intervention characteristics.

**Author and year**	**Why**	**What**	**How**	**Who**	**How much, when, and where**			**Tailoring and modifications**	**How well**
	**Therapeutic frameworks**	**Techniques**	**Overall process**	**Therapist qualification**	**No. of participants in the group & therapist to client ratio**	**Dosage**	**Settings**		**Fidelity assessment**
Aithal (2020)	Integrative therapeutic framework consisting of 8 principles informed by humanistic, psychodynamic and developmental theories	• Mirroring • Sensorimotor-based activities (Scharoun et al., 2014) • Sherborne developmental movement (Weston, 2012) • Imaginative role play (Westby, 2013)	4 modules, 10 semi structured sessions starting with a ritual followed by warm up, theme exploration and closing ritual	A licenced DMP practitioner	3 to 6 children, one therapist and one co-facilitator	DMP, 10 group sessions, 40 min twice a week	Two special educational needs setting	Yes. Depending on the needs of the group	Three evaluators rated video recordings of sessions (3, 6, and 9). Results indicated 75% and above adherence to the protocol
Sengupta and Banerjee (2020)	Not reported	• Mirroring • Bartenieff fundamentals • Comfort touch • Improvisation	24 sessions (45 min/per session) over a 3-month period	Researcher trained in DMP	One to one	DMP, 24 sessions (45 min/per session) over a 3-month period	Special School	Not reported	**NA**
Athanasiadou and Karkou (2017)	• Persons centred (Rogers, 1967) • Chace interactive model of DMP (Chaiklin and Schmais, 1986) • Intersubjectivity theory (Stern, 2005; Trevarthen, 2005; Meltzoff and Brooks, 2007) • Kinaesthetic empathy (Berger, 1972) • Sherborne Developmental Movement (Sherborne, 2001) • Embodiment-Projection-Role model (Jennings, 1999)	• Sensorimotor-based activities (Scharoun et al., 2014). • Mirroring (Wengrower, 2010) • Moving in Synchrony • Purposeful Misattunement (Stern, 1985) • Use of props • Use of metaphors • Rhythmic movement explorations • Embodied play, symbolic play activities • Shaping circle and moving in or away • Relaxation play • Goodbye movements	**Intervention programme structure**: Four modules- with eight sessions divided unevenly (module 1- one session, module 2- three sessions, module 3 and 4- two each) **Session structure:** Loosely structured around warm up mid-face and closure.	A licenced DMP practitioner	3 children and 1 adult	DMP, 8 group sessions, 50 min once a week	Special School	• **Case 1:** • Therapeutic Holding Environment (Winnicott and Rodnam, 2005) • Sensorimotor-based activities (Scharoun et al., 2014). • **Case 2:** • Mirroring (Wengrower, 2010) • **Case 3:** • Purposeful Misattunement (Stern, 1985)	**NA**
Devereaux (2017)	• Child-centred approach (Rogers, 1967) • Social engagement theories (Greenspan and Wieder, 1999) • Relational interaction (Ogden et al., 2006)	• Synchronistic rhythmic action • Self-expression movements • Building connexion on movement level • by moving closer, reaching • Circle formation • Tapping, stretching movements • Expansive movements • Understanding, reflecting, expanding non-verbal expressions • Attuned improvisation • Use of music • Use of props • Relaxation techniques	**Session structure**: Warm-up • Theme development • Closure portion	Registered or Board certified DMP	5–7 children and 1 or 2 adults	DMP, 30 Min, Once a Week	Special Education	1 child with Down's syndrome, 1 child with Cerebral palsy	**NA**
Houghton and Beebe (2016)	Literature review refers to • Disruption and repair (Beebe and Lachmann, 1994) • Dyadic systems view (Beebe and Stern, 1977) • Laban Movement Analysis (Laban, 1956; Bartenieff and Lewis, 1980)	• Interpersonal coordination • Imitation • Mirroring or Attunement • Synchrony • Use of kinesphere, different planes • Following the child's lead in the movement patterns, energy level,	**Session structure:** Unstructured movement exploration	Final year master's DMP training at a program approved by ADTA	One child and one adult	DMP, 30 Min, one session	Multipurpose special school	Beginning- Movements directed as he was not able to follow and imitate later turned into mirroring	
	• Kestenberg Movement Profile (Amighi et al., 2018)	• amount of connexion, direction • Intuitive and improvisational exploration							
Chiang et al. (2016)	Creative movement play approach was developed using: • Joint Engagement (Kasari et al., 2010) • Body informed intersubjectivity (Samaritter and Payne, 2013; Lee, 2014) • Child-centred approach (Rogers, 1967)	• Imitation • Mirroring • Toy play • Movement play • Meaningful play routine • Facilitating sharing communication • Encouraging child's initiating communication • Managing child's emotional regulation	**Intervention programmes structure:** 10 modules with different objectives and overall process **Session structure:** • Reviewing the dyad homework • coaching effective caregiver-child interaction • Discussing handout on objectives, goals of the homework	Two licenced clinical psychologists and one licenced dance/movement intervention	Parent child dyad and each interventionist worked with 4 to 7 dyads separately	DMP, 20 sessions, 60 min, Twice a Week across 2 months	Clinical & research setting	Movement play routineAffective attunementFacilitate Joint Engagement State	2 independent evaluators for videotapes of pre, post 3 months follow up- Therapists- high internal reliability Cronbach's α=0.96 Parents- moderate internal reliability
Samaritter (2015)	Literature review referred to-Interpersonal engagement theories from social cognition, social neuroscience, developmental perspectives • Theory of Mind (Baron-Cohen et al., 1985) • The weak central coherence theory (Frith, 1989) • Executive functioning theory (Ozonoff et al., 1991)	• Open-ended movement explorations • Use of props and variation of movement actions as starting point of improvisation • Structured games for example martial arts forms, baseball, dodge ball actions of synchronisation, attunement and dyadic engagement • Witnessing Mode- Space for individual movement experience • Joint movement- creating sameness • Movement Dialogue—Sharing sameness and otherness • Other relational modes- stillness, short oral evaluations	**Session structure:** Opening • Warming up • Structured games and dance/movement activities • Open movement activities • Closure	Researcher as Therapist (Qualified DMP Therapist)	One child and one adult	Shared Movement Approach (SMA) Intervention; 12 sessions,	Dutch Mental Health Care Centre (Clinical Outpatient Therapy)	**NA**	**NA**
Wengrower (2010)	Literature review referred to • Psychodynamic-Developmental (Alvarez, 1996) • Developmental individual difference relationship-based model (DIR) (Greenspan and Wieder, 2006) • Laban Movement Analysis (Laban, 1956; Bartenieff and Lewis, 1980) • Kestenberg Movement Profile (Amighi et al., 2018)	• Imitation (Stern, 1985) • Mirroring (Loman, 1998) • Attunement (Loman, 1998) • Empathic reflection (Sandel, 1993) • Use of transitional objects (Winnicott, 1971) • Exploratory movements and play • Sharing rhythm • Ritualistic actions	**Session structure:** Details not described explicitly • Warm up and movement dialogue are mentioned in fifth session	Qualified DMP Practioner	One child one adult	NA	Special education settings	**NA**	**NA**
Hartshorn et al. (2001)	Literature review referred to • Physiology of stress reduction and enhancing attentiveness (Field, 1998)	• Hello song • Clapping the syllables • Use of props- hoops, gym mats, tambourine, stickers • Jumping in and out of hoops, obstacle course, making different shapes, start and stop games • Behavioural class management techniques	**Session structure:** Warm up activity • Intermediary activities structured with task, role and space • Cool down	Trained movement Therapists.	3 to 8 children and 2 adults	DMP, 16 sessions, 30 min, Twice a Week	School for children with Autism	**NA**	**NA**
**Summary**	• Not all studies have mentioned the intervention approach explicitly • Person-centred approach, • Social engagement and intersubjectivity related theories are most common	• Mirroring-most common • Sensorimotor explorations creatively merged alongside the use of play techniques, rhythm and props	• Overall structure described by only two studies • Session structure: • Semi-structured most common	• Qualified DMP • Experience of the therapist mentioned in only one study • Three studies with researcher as therapist	• Four studies with one to one DMP • Three studies group sessions DMP • One study with caregiver-mediation	• Frequency: Once or twice a week • Total: 8 to 20 sessions across-One and a half to 2 months • Duration-30 to 60 min	7 studies in special educational needs settings	No clear pattern- driven by the needs of each child	Mentioned by two studies

#### Therapeutic Frameworks

This section considered the theories, principles and approaches that conceptually informed the DMP intervention. It was noticed that reporting the type of therapeutic approach adopted was not a widespread practice among DMP researchers as only four studies specifically described their approach and lens in which the therapy sessions were delivered (Chiang et al., 2016; Athanasiadou and Karkou, 2017; Devereaux, 2017; Aithal, 2020). The rest of the studies only reported either the structure of the session or the activities conducted during the DMP sessions. Although all nine studies included in the review have referred to various theories in their literature review section, it is unclear if those theories have really influenced the development and delivery of the DMP sessions or if they were referred to from a research perspective. However, for better understanding of the subject, the data extraction process of the present review considered all important theories mentioned in the studies' literature review as well as in their methods sections, distinguishing between the two.

As shown in Table 3, the four studies that were explicit about their therapeutic approach referred to person-centred or humanistic principles^2^ (Chiang et al., 2016; Athanasiadou and Karkou, 2017; Devereaux, 2017) and integrative approach (Aithal, 2020). In a humanistic approach of DMP, the focus is generally on strengthening clients' resources in the here-and-now (Karkou and Sanderson, 2006). For the rest of the studies, details provided within the description of sessions and the literature review appear to have influences from the humanistic approach. For instance, in studies Wengrower (2010), Samaritter (2015), Houghton and Beebe (2016) references are made to kinaesthetic empathy, Chace interactive model of DMP (Chaiklin and Schmais, 1986), initiating the movements following the child's lead in the movement patterns and energy level resonate with the humanistic principles.

Apart from the humanistic approach, elements of developmental ideas^3^, psychodynamic^4^, and behavioural^5^ thinking were also found in some of these studies. Four studies (Wengrower, 2010; Samaritter, 2015; Athanasiadou and Karkou, 2017; Aithal, 2020) have referred to the relevance of developmental models such as Developmental Individual difference Relationship-based model (DIR) (Greenspan and Wieder, 2006) and Sherborne Developmental Movement (Sherborne, 2001) as relevant to DMP sessions. These developmental approaches allowed the therapists to determine movements, to engage with sensorimotor explorations, to support fundamental capacities for joint attention, to achieve regulation, and encourage children's development of a wide range of emotional, social and communicative skills appropriate to the stages of development and age. Traces of psychodynamic perspectives were found in three studies (Wengrower, 2010; Houghton and Beebe, 2016; Aithal, 2020). A dyadic system view of communication (Beebe and Stern, 1977) and the psychodynamic-developmental model (Alvarez, 1996) mentioned in the latter two studies elucidate the nature of interpersonal process and interactive regulation in the dyad. Wengrower (2010), Houghton and Beebe (2016) have brought in psychodynamic thinking by viewing the movement interaction from mother-child lens and attachment patterns. Influences from behavioural approaches were also found in two quantitative studies (Hartshorn et al., 2001; Chiang et al., 2016). These studies focused on how behaviours and skills change, the way learning takes place and also stressed the role the environment plays in enabling new learning within the context of DMP sessions.

The most common trend prevalent in seven out of the nine studies was the allusion to theories focusing on social engagement and interpersonal relationships. As shown in Table 3, eleven different theories have been reported to describe processes that are often associated with achieving: shared understanding, relating one situation to another, broad range of social roles and relationships, interaction between individuals and their environments from social cognition, social neuroscience and also from a developmental perspective. Among those eleven theories, the intersubjectivity theory (Stern, 2005; Trevarthen, 2005; Meltzoff and Brooks, 2007) used for describing relations between people, has influenced the development of three DMP intervention models called Shared Movement Approach (SMA) by Samaritter (2015); Creative Movement Play approach by Chiang et al. (2016) and See-Saw by Aithal (2020). Intersubjectivity theory was also used to explain a core concept used in DMP called Kinaesthetic empathy (Jerak et al., 2018). Further, the same theory has encouraged Houghton and Beebe (2016) to look more deeply at disruption and repair within a dyadic system.

Within these different relational theories, four studies (Wengrower, 2010; Samaritter, 2015; Houghton and Beebe, 2016; Sengupta and Banerjee, 2020) have incorporated movement-based systems such as Laban Movement Analysis (Laban, 1956; Bartenieff and Lewis, 1980) and Kestenberg Movement Profile (Amighi et al., 1999) to analyze as well as to create movement-based activities. DMP sessions widely incorporate play within movement activities. Three studies have explicitly mentioned the application of theories related to play in general (Chiang et al., 2016; Aithal, 2020) and specifically; Athanasiadou and Karkou (2017) refer to a dramatherapy model called Embodiment-Projection-Role (Jennings, 1999). Overall, person-centred and developmental approaches with suffuse and overlap of social engagement, intersubjectivity theories and play theories were found to be most prevailing in DMP intervention for children with ASD.

#### Techniques

The focus here was on what practically happened during DMP sessions. The studies have consistently mentioned mirroring as one of the basic techniques. Many different terms referring to similar concepts have been used across these studies as a way of improving interaction: attunement; understanding, reflecting, imitation, expanding non-verbal expressions leading to attuned improvisations, affective synchrony, movement synchrony, movement coordination, relational knowing, shared movement, reciprocal responsiveness/interaction, and many more. In some instances, these terms have been used synonymously while some authors have distinguished between them. Wengrower's (2010) study investigated the difference between imitation and mirroring and found that the major difference between the two was in the quality of interaction. Unlike imitation, Wengrower (2010) argued that mirroring involves the therapist making similar (and not identical) body movements reflecting the essence of the movement of the client which are either coordinated in time or with a slight echo (Fitzpatrick, 2018). The assumption is that the moving partners experience in their own body the qualities of each other's movements to experience motor resonance and perceive better emotional understanding of each other leading to somatic relationship by connecting with attunement. In the past, the term attunement has been described as a component of mirroring that often goes beyond empathy and can be seen as a product of mirroring (Erskine and Trautmann, 1997; Trevarthen and Fresquez, 2015). The process of attunement is reported to have two levels (Jerak et al., 2018). At first, the moving partners are fully aware of the other person's sensations, needs, or feelings and the next step is the communication of that awareness (Jerak et al., 2018). Tortora (2010) identifies three subcategories in mirroring: modified mirroring, mirroring exaggerated, and mirroring diminished. In addition, misattunement, disruptions and purposeful misattunement have also been reported as part of the process and occasionally as useful techniques for regulating and introducing new movement vocabulary to children with ASD (Houghton and Beebe, 2016; Athanasiadou and Karkou, 2017).

The next set of most popular techniques as mentioned in all seven studies were related to sensorimotor explorations creatively merged alongside the use of play techniques, rhythm and props. Sensorimotor-based activities (Scharoun et al., 2014) involved open-ended movement explorations and structured games. Importance was given to spontaneous movement interactions and expanding the children's movement vocabulary. Across the studies opportunities were offered for body part identification and awareness through stretching, tapping, movements from martial art, baseball, dodgeball actions, jumping in and out of hoops, obstacle course, making different shapes, start and stop games and many more (Hartshorn et al., 2001; Athanasiadou and Karkou, 2017; Devereaux, 2017; Aithal, 2020). All these movement explorations were reported to be used playfully and to be linked to different types of play such as embodied play, symbolic play, imaginative play, movement and rhythmic play, structured, and unstructured play activities across all seven reviewed studies.

In addition, the reviewed studies made use of props such as hoops, gym mats, tambourines, stickers, elastic bands, many other toys, and music. These props were used for different purposes such as self-expression, sensory stimulation, sensory integration and relaxation; they were also used as starting points in movement improvisation, as transitional objects and as concrete ways of connecting. Some of the studies reported incorporation of relaxation techniques (Hartshorn et al., 2001; Athanasiadou and Karkou, 2017; Devereaux, 2017) while the rest of the studies have merged relaxation techniques with the use of props and music. Laban movement vocabulary such as body, efforts, shape and space were used, along with body orientation, engagement and regulation. These elements were reported as important by many authors (Samaritter, 2015; Chiang et al., 2016; Houghton and Beebe, 2016; Athanasiadou and Karkou, 2017; Sengupta and Banerjee, 2020). Behavioural class management techniques appeared to be least popular as only one study mentioned them as part of the intervention (Hartshorn et al., 2001).

To sum up, particular attention was given to intuitive and improvisational exploration of movements with mirroring as the key technique in DMP sessions across all the studies.

#### Overall Process

This section explored the DMP intervention programme structures spreading across research projects and the structure within each session. Chiang et al. (2016), Aithal (2020) and Athanasiadou and Karkou (2017) were the only three studies to describe the overall structure. Chiang et al. (2016), included ten modules and each module consisted of two sessions targeting various objectives. Athanasiadou and Karkou (2017) described four modules with eight sessions divided unevenly (module 1: one session; module 2: three sessions; module 3 and 4: two each). Aithal (2020) described eight principles that informed four modules containing ten sessions. Houghton and Beebe (2016) reported that the intervention consisted of twenty sessions while Sengupta and Banerjee (2020) mentioned 24 sessions. However, further description of the course was not offered in those two studies.

With regards to the structure of each session, Hartshorn et al. (2001), Samaritter (2015), Athanasiadou and Karkou (2017), Devereaux (2017), and Aithal (2020), had similar session structures. The sessions began with a warm-up, moved to theme development and/or structured/unstructured play activities and closed with cooldown activities. Unlike these five studies, the session structure in Chiang et al. (2016), Houghton and Beebe (2016) studies appeared different. Chiang et al. (2016) showed traces of a behaviourist approach where each session consisted of reviewing the dyad homework film-taped by the parent, followed by effective caregiver-child interaction with guided practise, demonstration, modelling, and feedback. The one-to-one session described by Houghton and Beebe (2016) was completely unstructured. The session began with child-directed movements usually leading to mirroring and improvisational exploration. Wengrower's (2010) study did not mention anything about the session structure.

#### Dosage

As shown in Table 3, four studies involved individual therapy sessions while another four studies held group therapy sessions, all delivered by qualified dance movement therapists. Teaching assistants and care givers were also involved when groups were large (Hartshorn et al., 2001) and licenced psychologists were involved in the Taiwanese study (Chiang et al., 2016). In the latter study (Chiang et al., 2016) there was also parental involvement with parents receiving training in caregiver-child interaction. The number of participants in a group session varied from three to eight with an average of five children per group. Studies with group therapy were delivered over one and a half to 2 months and sessions ranged from 30 min (Hartshorn et al., 2001; Houghton and Beebe, 2016; Devereaux, 2017) to 60 min (Chiang et al., 2016). Sessions were delivered once or twice a week totalling from 8 (Athanasiadou and Karkou, 2017) to 24 sessions (Sengupta and Banerjee, 2020) as shown in Table 3. There was lack of clarity on the dose and intensity of the therapy in the studies where the focus was on specific sections of the process of therapy against the outcome (Wengrower, 2010; Houghton and Beebe, 2016). Moreover, there was no clear trend or pattern that was noted to indicate the relationship between length of therapy and the benefits gained by the client group. In general, DMP dosage was not always theoretically grounded or in accordance with the aims of the intervention. Rather it appeared to be driven by logistics and availability of funding.

### Research Question 2- Data-Collection Methods and Findings

This section synthesised information on how data were gathered and the results that were reported in the studies.

#### Data-Collection Methods

Qualitative, quantitative and artistic enquiry research methodology studies were considered. As shown in Table 2, video recording methods were used by the majority of the reviewed studies as it enabled particular sections of the session to be reviewed at a later point. For instance, Houghton and Beebe (2016) carried out a video micro analysis in real-time and in slow-motion to develop an extensive narration of the video clip, exploring key changes of the interpersonal movement sequences. Samaritter (2015) also used a retrospective video analysis where movement markers of interpersonal relating were coded based on Laban Movement Analysis (LMA) using ELAN software to develop an observation scale. Studies that used artistic inquiry as a research methodology, somatic responses and written reflections were complemented with video recordings of the sessions (Athanasiadou and Karkou, 2017; Aithal, 2020). The next popular approach after video recordings in these studies was collecting narratives and therapists' diaries (Wengrower, 2010; Houghton and Beebe, 2016; Athanasiadou and Karkou, 2017). Semi-structured interviews of the parents and educators have also been utilised in the qualitative studies included in this review (Devereaux, 2017).

Quantitative studies have relied upon movement or behavioural observations by trained movement observers with parameters such as duration and frequency of occurrence of target movements or behaviours; these observations produced numerical data (Hartshorn et al., 2001; Chiang et al., 2016). Standardised questionnaires and test batteries were employed in four studies (Samaritter, 2015; Chiang et al., 2016; Aithal, 2020; Sengupta and Banerjee, 2020). Samaritter (2015) was the only study to use self-reporting method as they had adolescent participants in their study. No tool was used more than once and hence the quantitative results were not suitable for meta-analysis. Overall, the preferred methods of data collection were through video observations and semi-structured interviews from clinicians, parents or educators' perspectives.

The reviewed studies addressed the contribution of DMP for children with ASD. Outcomes were grouped under the following domains based on literature themes:

- Social and communication: skills used to interact, both verbally and non-verbally to communicate messages, thoughts and feelings with others.
- Psychological (cognitive, emotional, & behavioural): skills necessary for bonding, self-regulatory behaviour, displaying emotions, empathy and to cope with challenges; combination of several critical brain functions related to memory, judgment, intuition, attention, concentration, ability to learn and process information.
- Physical and sensory: abilities related to the whole body in terms of endurance, stamina, flexibility, speed, coordination, balance, sensory inputs, and integration.

#### Social and Communication

Eight out of nine studies mentioned the effects of DMP on improving different social skills. Positive impact on awareness of personal boundaries, relationship with the therapist, entering group relationship, understanding of social dynamics and social relatedness were noted in many studies (Samaritter, 2015; Houghton and Beebe, 2016; Athanasiadou and Karkou, 2017; Devereaux, 2017). Significant improvement in Social Engagement and Attunement Movement (SEAM) observation scale and scores obtained on a social questionnaire administered pre-post therapy in Samaritter (2015) thesis. In Aithal (2020) study, a statistically significant result on the social communication questionnaire was observed in the intervention group irrespective of whether they preferred verbal or non-verbal mode of communication. The measurement of social behaviours through questionnaires and self-report indicated that improvement was not limited just to the therapeutic setting; instead, the participants were able to generalise it to their real life as well.

All qualitative and arts-based studies reported progress in overall communication (verbal and non-verbal). As non-verbal communication is predominantly used in DMP sessions, therapists have observed improvement in expressive and receptive oral vocabulary (Houghton and Beebe, 2016; Athanasiadou and Karkou, 2017; Aithal, 2020). It was cited that DMP provided opportunities for an increase in movement vocabulary (Samaritter, 2015; Athanasiadou and Karkou, 2017; Sengupta and Banerjee, 2020). In turn it provided scope for experiencing group dynamics and different levels of communication (Athanasiadou and Karkou, 2017). Case studies have reported that the children who did not show communicative intent in the beginning of the therapy improved to such a level where they initiated conversation by greeting the therapist (Houghton and Beebe, 2016). As a whole, qualitative, quantitative and arts-based studies suggest that DMP can play a significant role in improving different aspects under the social domain in children with ASD.

#### Psychological (Emotional, Behavioural, and Cognitive)

Parents, educators and therapists have noted progress in emotional regulation (Athanasiadou and Karkou, 2017; Devereaux, 2017; Aithal, 2020). Children improved in their ability to modify their emotional reactions. The coping mechanisms were enhanced as they had better control over their movements to increase or decrease the intensity of the movement. It was evident that there was improvement in awareness (self and others). It has also been reported that participants presented a better mood for the rest of the day after sessions (Athanasiadou and Karkou, 2017; Devereaux, 2017). Studies report that the participants improved in attention, concentration, on task passive behaviour and also on transition from one activity to another (Hartshorn et al., 2001; Devereaux, 2017). Statistical tests in Hartshorn et al.'s (2001) study revealed a reduction in the time that the children wandered in the room, showing that they had developed better abilities to focus. On task active behaviour and joint attention did not show any significant enhancement after therapy. In Chiang et al.'s (2016) study, the different types of joint engagement (JE) states between parent and child were studied. Improvements were seen only at the 3 months follow up stage in unengaged JE, child initiated supported JE and child initiated co-ordinated JE. No statistically significant difference was seen in parent initiated JE states at post treatment and follow up assessments. Only in Devereaux's (2017) study academic engagement has been reported. The teachers interviewed in this study reported that the DMP sessions facilitated transition into academic activities. It was also reported that children performed better in class after the session as their energy had been channelled. This helped them to sit and focus during the lessons.

#### Physical and Sensory

Qualitative and arts-based studies (Athanasiadou and Karkou, 2017; Devereaux, 2017) described reductions in the self-stimulatory and stereotypical behaviours. The children appeared more relaxed and calmer. However, Hartshorn et al.'s (2001) study did not find any statistically significant reduction in stereotypical behaviours.

In summary, various tools have been used to examine the contribution of DMP for children with ASD. Most frequently occurring outcomes fell under the social domain followed by cognitive, emotional and physical. There was only one study which mentioned academic engagement which again overlaps with cognitive, physical and sensory domains.

### Results of Quality Assessment

The methodological quality of the studies are heterogenous as per the MMAT (Hong et al., 2018) appraisal tool as shown in Table 4. Six studies were assessed as level 4 evidence and there was one study at level 2B and two studies at 2C levels as per the criteria from the Centre for Evidence Based Medicine (March, 2009). Two qualitative studies, one quantitative study and two mixed methods study have addressed at least four out of five questions on the MMAT quality assessment (Samaritter, 2015; Chiang et al., 2016; Athanasiadou and Karkou, 2017; Devereaux, 2017; Aithal, 2020). However, limitations were observed in most of the studies. For the below research aspects were inadequately addressed: intervention characteristics, methodological pitfalls, challenges at the stage of recruitment and implementation of interventions, attrition rates, sufficient correlation between theory and outcomes. One of the major drawbacks identified in Wengrower (2010) and Sengupta and Banerjee (2020) was the lack of a clear link between data sources, collection, analysis and interpretation. Further, there were some observations in the narration of the case studies that were not always substantiated with adequate data (Wengrower, 2010; Houghton and Beebe, 2016; Athanasiadou and Karkou, 2017; Sengupta and Banerjee, 2020).

**Table 4:** Quality appraisal of the studies using the mixed methods appraisal tool (MMAT).

**MMAT category of study designs**	**Study**	**Responses**					**MMAT methodological quality criteria (Hong et al., 2018)**
Qualitative		**1.1**	**1.2**	**1.3**	**1.4**	**1.5**	1.1. Is the qualitative approach appropriate to answer the research question?
	Athanasiadou and Karkou (2017)	Y	Y	Y	C	Y	1.2. Are the qualitative data collection methods adequate to address the research question?
	Devereaux (2017)	Y	Y	Y	Y	Y	1.3. Are the findings adequately derived from the data?
	Houghton and Beebe (2016)	Y	C	Y	C	Y	1.4. Is the interpretation of results sufficiently substantiated by data?
	Wengrower (2010)	Y	N	N	N	N	1.5. Is there coherence between qualitative data sources, collection, analysis and interpretation?
Quantitative non-randomised		**3.1**	**3.2**	**3.3**	**3.4**	**3.5**	3.1. Are the participants representative of the target population?
	Hartshorn et al. (2001)	Y	C	N	N	C	3.2. Are measurements appropriate regarding both the outcome and intervention (or exposure)?
	Chiang et al. (2016)	Y	Y	Y	Y	Y	3.3. Are there complete outcome data? 3.4. Are the confounders accounted for in the design and analysis? 3.5. During the study period, is the intervention administered (or exposure occurred) as intended?
Quantitative		**4.1**	**4.2**	**4.3**	**4.4**	**4.5**	4.1. Is the sampling strategy relevant to address the research question?
descriptive	Sengupta and Banerjee (2020)	N	Y	N	Y	N	4.2. Is the sample representative of the target population? 4.3. Are the measurements appropriate? 4.4. Is the risk of non-response bias low? 4.5. Is the statistical analysis appropriate to answer the research question?
Mixed methods		**5.1**	**5.2**	**5.3**	**5.4**	**5.5**	5.1. Is there an adequate rationale for using a mixed methods design to address the research question?
	Samaritter (2015)	C	Y	Y	Y	Y	5.2. Are the different components of the study effectively integrated to answer the research question?
	Aithal (2020)	Y	Y	Y	Y	Y	5.3. Are the outputs of the integration of qualitative and quantitative components adequately interpreted? 5.4. Are divergences and inconsistencies between quantitative and qualitative results adequately addressed? 5.5. Do the different components of the study adhere to the quality criteria of each tradition of the methods involved?

*Y, Yes; N, No; C, Can't tell*.

The lack of clarity in reporting methodological procedures have affected the trustworthiness of many studies (Hartshorn et al., 2001; Wengrower, 2010; Houghton and Beebe, 2016; Sengupta and Banerjee, 2020). As a result of insufficient reporting of children's demographic characteristics and contextual background information, it was unclear whether the findings are transferable (Wengrower, 2010; Samaritter, 2015; Houghton and Beebe, 2016; Sengupta and Banerjee, 2020). Poor reporting of DMP intervention in three studies made it difficult to extract clear patterns of evidence (Hartshorn et al., 2001; Wengrower, 2010; Houghton and Beebe, 2016).

The two non-RCT quantitative studies (Hartshorn et al., 2001; Chiang et al., 2016) used age and level matched controlled groups and statistical testing of variables. They were identified with a high risk of bias as the recruitment process lacked randomisation. In Hartshorn et al.'s (2001) study the attrition rate and dropping out of participants were not mentioned and this might have skewed the outcomes to some extent. Aithal (2020) a mixed methods, study, was the only one to include randomisation of the participants, report attrition, use intention-to-treat analysis and report all the measured outcomes. However, the study did not include blinding of the researchers and participants and therefore it has a high risk for detection bias. Whilst as for all types of psychotherapy, it is difficult to blind participants to the type of intervention, it appeared that in Chiang et al. (2016) and Hartshorn et al. (2001) there were opportunities to blind for the outcome assessment. Hartshorn et al.'s (2001) study mentions that psychology graduate students rated the video. However, the description did not mention if they were blinded on the group information. Similarly, in Chiang et al.'s (2016) study, it is unclear if the clinicians administering the interviews and tests were aware of whether the participants were allocated to the control or the experimental group. This study introduced blinding while testing the fidelity of the interventionist to the treatment protocol, but there was incomplete information and reporting of the findings from other assessment tools used in the study.

## Discussion of the Systematic Review Findings

This review gathered clinical procedures and research findings from nine studies on DMP with children with ASD involving a total of 133 participants. The number of studies included remained small with heterogeneous outcome measures and compromised quality. There was only one mixed methods study (Aithal, 2020) with a randomisation component found during the literature search and only nine studies met the inclusion criteria that was very broad. It is frequently argued in DMP that RCTs alone cannot capture therapeutic processes as the creative arts therapies emphasise creativity and subjective ways of knowing (Junge and Linesch, 1993). However, there were only two studies with artistic inquiry (Athanasiadou and Karkou, 2017; Aithal, 2020) which met the inclusion criteria. The small number of studies meeting the inclusion criteria reflects the dearth of research work in the field.

The synthesis of data relating to the first research question (how do dance movement psychotherapists work with children with ASD?) revealed that humanistic and developmental approaches delivered through semi-structured sessions using play-based sensorimotor activities and mirroring techniques are the most common ways of working with children with ASD. These approaches are in agreement with Nind (1999) who supported the need for interventions with minimal instructions or teacher direction, and more dependent on intuitive responses. The approaches and techniques are on a par with case reports, documentaries and reports by pioneers in DMP such as Adler (1968), Siegel (1973), Kalish (1977), Erfer (1995), and Loman (1995) in propounding the body-informed and non-verbal interpersonal approaches that attempt to meet the children at the level they are and to facilitate expressive relationship with the environment. Behavioural theories are minimally referred to in DMP which is noteworthy especially given the client population and the prevalence of behavioural thinking in existing literature (Pierce and Cheney, 2017). It is possible that DMP is indeed offering a new approach that complements existing interventions.

There are similarities in the DMP approaches across the age range of ASD population. Marchant et al. (2018) in their systematic review on DMP with adults with ASD, synthesised that person-centred approach with techniques such as mirroring, Baum Circles, sensory integration, synchronisation, six-part storey making, dyadic leading and following, as well as moving together, breath work and relaxation exercises, props and verbal processing. The differences in the approaches for the younger population with ASD were the use of developmentally appropriate play-based activities alongside other DMP techniques with the focus on joint attention and other cognitive prerequisites for communication. Marchant et al. (2018) also reports that the studies reviewed were predominately structured with the exception of Mateos-Moreno and Atencia-Doña (2013) and Edwards (2015) who pertained to a semi- or un-structured framework. While in this review, the majority of studies have preferred semi-structured DMP sessions over fully structured or unstructured sessions. It can be deduced that DMP group sessions for children are most likely to be semi-structured for children with ASD and unstructured sessions are feasible while working on one-to-one. Both group and individual sessions appeared to be popular while working with children on the autism spectrum. However, there was no clarity and correlation between the severity of ASD and the type of session nor there was a clear trend between duration, frequency, intensity and progress made by children. One of the most relevant result of this review is that only three studies have reported on complete DMP intervention programme structure. Poor research reporting of the sessions has created several gaps in interpretation of the findings.

In terms of settings, special education settings were the most common settings across the studies. Advantages of conducting studies within special education settings could be that attrition is minimised and there are more opportunities for consistent observations from different perspectives. Similar advantages have been reported in studies involving adults with ASD conducted in education institutions or specialist centres (Marchant et al., 2018).

With regards to the second research question (How do different studies examine the effectiveness and processes involved in DMP interventions? What are their findings?), the search results were in accordance with Vulcan's (2016) claim that the research available in relation to children with ASD often leans toward case studies. Although the studies included in the current systematic review revealed some positive outcomes, these results cannot be generalised since included studies were placed at the lower and intermediate level of evidence with varying methodological quality. This led to high heterogeneity of the results, unconvincing evidence, and exertion in recognising key results. Since the studies did not have consensus in terms of the parameters measured, tools used and the output, there was a risk of *mixing apples and oranges* (Higgins and Green, 2008) leading to meaningless results if meta-analysis was performed. Hence, the outcomes were synthesised and mapped under broader domains, namely social and communication, psychological and physical/sensory.

Improving social skill was a major area of interest in the reviewed studies; a substantial evidence reports that DMP is potentially able to enable the development of relationships. This extensive interest stands as per the NICE guidelines (NICE, 2016) that value interventions that address the social-communication core features of ASD. Despite considerable extent of importance given to the core features of ASD in the reviewed articles, some of the claims were not fully substantiated with data. For example, serotonin levels, EEG activity, sensory motor mirroring and many others were proposed as probable reasons for change in social skills without linking them with data and appropriate tools for measurement. Hence, further explorations are required to look at the underlying factors bringing changes in the client group.

NICE guidelines (NICE, 2016) for children with ASD also highlight the importance of managing co-existing emotional issues leading to anxiety and depression in addition to cognitive areas such as increasing joint attention, joint engagement, and on-task behaviours through play-based strategies. The findings from the present review on the role of DMP in improving emotional regulation, awareness and anxiety coping strategies as perceived by parents, educators and therapists are promising. In contrast, findings relating to the development of cognitive skills and physical/sensory outcomes remained inconsistent, calling for further clarification. For instance, the findings on task behaviour and joint attention did not show any significant enhancement after therapy in quantitative studies while qualitative studies did observe progress. In addition, Chiang et al.'s (2016) study found improvements stage in unengaged joint engagement, child initiated supported JE and child initiated co-ordinated JE only at 3 months follow up and not immediately after the intervention. But the reasons for these inconsistencies are not clear.

Similar issues where findings from qualitative and quantitative findings contradicted each other were noticed in other domains as well. For example, improvements from brief moments of eye contact to sustained and meaningful eye contacts with the therapist (Houghton and Beebe, 2016; Athanasiadou and Karkou, 2017) and with the group members (Athanasiadou and Karkou, 2017) have been reported. By contrast, Hartshorn et al.'s (2001) quantitative observation on eye contact does not report statistically significant improvement. This could possibly be because of the nature of assessment and methodology of the research studies. In a natural context, eye contact varies within the content and meaning of the situation. Probably, quantifying the progress in terms of duration of the behaviours or actions sustained may not always reflect as the correct measurement of change. As a result, gaps in understanding the appropriate dosage, follow up findings and type of assessment tools that are valid and sensitive to pick up changes will need to be addressed for better clarity.

To compare the results of the present review with the other studies on the same topic, not many reviews were found in DMP and there was no review on this topic specifically focusing on children below 16 years. The current results were compared with findings of the reviews by Koch et al. (2014) and Scharoun et al. (2014). Koch et al. (2014) studied health-related psychological outcomes of DMP and reported DMP was as effective evidence-based intervention for children and adults with ASD. Similarly, the study by Scharoun et al. (2014) identified the success of DMP interventions in both individual and group settings for people with ASD. Unlike these two reviews, the present study does not share the confidence that DMP is an effective intervention for this client population due to the high risk of bias of the reviewed studies, extensive variability in methodology, limited and inconsistent usage of valid, standard tools for assessments and dearth of replicable outcomes. However, this study does acknowledge the *potential* in DMP to be an effective intervention for children with ASD.

To sum up the key contributions of DMP, social and emotional domains stand out among different parameters while communication and sensory domains are interwoven with the other domains. Improvements in core issues of ASD and comorbid problems such as making connexions, awareness of self and others, emotional regulation, joint engagement, repetitive movements have been frequently reported. There is lack of clarity in the underlying factors that might have brought these changes. Furthermore, these findings are inconclusive due to the small sample sizes of the studies included in the review. Generalising the outcomes to the population is not, therefore, possible leading to limited evidence on the contribution of DMP to children with ASD. Overall, there were issues with the quality for the studies reviewed. It is necessary for the authors to adhere to the reporting guidelines to enhance transparency and the impact of the interventions (Hoffmann et al., 2014). Although the findings of this review are inconclusive, they clearly highlight gaps in existing literature which need to be dealt with in depth for future developments in research, policy and practise.

### Potential Bias in the Review Process

This review includes the doctoral thesis of the first author (Aithal, 2020) and the co-authors who were part of the supervisory team. This potential source of bias has been addressed by involving an external researcher who was not part of the doctoral research team. In addition, the members in the research team are from diverse backgrounds which could potentially reduce bias by bringing in different perspectives and expertise.

Some of the limitations of the review were that unpublished studies and research articles in languages other than English were not included due to time and resource restraints. This could have led to a potential risk of bias. Furthermore, a funnel plot analysis designed to check for the existence of publication bias in systematic reviews was not possible due to low number of published studies included (Higgins and Green, 2011). One more issue influencing the external validity of this review could be the PICOS inclusion criteria set to identify studies. The way DMP was defined for the purposed review i.e., with a clear psychotherapeutic process and intent has limited the included studies. For example, the study by Ramachandran and Seckel (2011, p. 151), which outlined the basis of “synchronised DMP to simulate mirror neurons” was excluded because the authors described a DMP practise that parted from a conventional approach: children with ASD were invited to look into a room of mirrors, all located at varying angles to facilitate numerous allocentric views (Fidalgo and Martin, 2016).

## Summary of the Review

The systematic review suggests that DMP can potentially promote various aspects of well-being in children with ASD; however, evidence for its effectiveness remains inconclusive. There is a need for large sample 1B level studies (Burns et al., 2011) that use standardised and validated tools for evaluation which are appropriate for the population. The review also identifies limited evidence on long-term effects of DMP. Therefore, follow-up studies which assess outcomes at regular intervals after terminating the intervention are needed. Researchers should also consider including economic analyses and acceptability measures as they can provide a more realistic picture for practise implications and can connect research with policy, training and advocacy (Uttley et al., 2015). Additional needs to be given to setting the inclusion criteria on severity and comorbidity of ASD. Future studies could also consider exploring the relationship between particular approaches of DMP and diverse severity of ASD. Individual v/s group interventions for children with ASD also need to be researched so as to support clinical guidelines that take these issues into account. Overall quality and quantity of studies must grow markedly in this topic to make a substantial shift in what we know so far. Well-designed, detailed studies on the impact of DMP for children with ASD are warranted. Before well-designed RCTs are conducted and given the limited explanations of the key therapeutic factors that bring change, attention should be given to further understanding the therapeutic process. It was inferred from the review that qualitative and arts-based research designs that focus on the therapeutic process could be useful with regards to this issue.

## Data Availability Statement

The secondary data supporting the conclusions of this article will be made available by the authors without undue reservations upon request.

## Author Contributions

SA was responsible for organising, analysing, and writing up the current paper. ZM completed the systematic search with SA and VK. She also contributed to the editing of the text. VK guided and provided corrections for review. She also acted as a referee during the searches. SM, JP, and TK contributed to the development of protocol, revisions, and edits of the paper. All authors contributed to the article and approved the submitted version.

## Funding

This study was supported by Edge Hill University's Graduate Teaching Assistant Scheme.

## Conflict of Interest

The authors declare that the research was conducted in the absence of any commercial or financial relationships that could be construed as a potential conflict of interest.

## Publisher's Note

All claims expressed in this article are solely those of the authors and do not necessarily represent those of their affiliated organizations, or those of the publisher, the editors and the reviewers. Any product that may be evaluated in this article, or claim that may be made by its manufacturer, is not guaranteed or endorsed by the publisher.

## Footnotes

^1^Other titles known for the discipline internationally: dance movement therapy, dance-movement therapy, dance therapy, dance psychotherapy, movement therapy, and movement psychotherapy.

^2^“Humanistic” is an umbrella term used to refer to a number of different approaches to therapy (client/person-centred therapy is one such example–Rogers, 1942), all of which focus on strengthening clients' resources in the here-and-now, value I-thou relationships, empathy and consider the client as leading the process of the work (Karkou and Sanderson, 2006).

^3^Developmental perspectives focus on providing interventions that are developmentally suitable to the skills and capacities of participants (Karkou and Sanderson, 2006).

^4^“Psychodynamic” is a broad term which includes Freudian and meta-Freudian schools of thought, all of which believe in the existence of the unconscious, assume one's first years of life shape one's emotional life and attempt to make links with past experiences (Karkou and Sanderson, 2006).

^5^A behavioural approach believes that all behaviours are learned through interaction with the environment and focuses on establishing observable stimulus-response behaviours with clear expectations of behaviours to specific stimuli (Barbera and Rasmussen, 2007).
